# Raman Evaluation of the Crystallinity Degree of Star-Shaped Lactide Oligomers: An Influence of the Number, Length and Enantiomeric Composition of the Arms

**DOI:** 10.3390/polym18182310

**Published:** 2026-09-21

**Authors:** Alyona N. Bortsova, Liudmila Yu. Kozlova, Sergey M. Kuznetsov, Alexander A. Puchkov, Sergei O. Liubimovskii, Artem V. Bakirov, Nikita G. Sedush, Sergey N. Chvalun, Maksim N. Moskovskiy, Gulnara Yu. Nikolaeva, Sergey V. Gudkov, Alexey S. Dorokhov, Andrey Yu. Izmailov, Vasiliy S. Novikov

**Affiliations:** 1 Prokhorov General Physics Institute of the Russian Academy of Sciences, 119991 Moscow, Russia; alenka.selihova3@gmail.com (A.N.B.); lus.kozlowa2011@kapella.gpi.ru (L.Y.K.); kuznetsovsm@kapella.gpi.ru (S.M.K.); liubimovskii@kapella.gpi.ru (S.O.L.); nikolaeva@kapella.gpi.ru (G.Y.N.); vasiliy1992@gmail.com (V.S.N.); 2 MT2 Department, Bauman Moscow State Technical University (BMSTU), 105005 Moscow, Russia; 3 Enikolopov Institute of Synthetic Polymeric Materials of the Russian Academy of Sciences, 117393 Moscow, Russia; puchkov1208@gmail.com (A.A.P.); bakirov.artem@gmail.com (A.V.B.); nsedush@gmail.com (N.G.S.); s-chvalun@yandex.ru (S.N.C.); 4 National Research Center “Kurchatov Institute”, 123182 Moscow, Russia; 5 Federal Scientific Agroengineering Center VIM, 109428 Moscow, Russia; maxmoskovsky74@yandex.ru (M.N.M.); dorokhov.vim@yadex.ru (A.S.D.); vim@vim.ru (A.Y.I.); 6 Department of Fundamental Sciences, Bauman Moscow State Technical University (BMSTU), 105005 Moscow, Russia

**Keywords:** Raman spectroscopy, lactide, polylactide, star-shaped oligomers, branched oligomers, molecular architecture, crystallinity degree

## Abstract

The control of structure-dependent properties of materials widely applied in the manufacture of medical products is extremely important. The purpose of this study was the Raman evaluation of the crystallinity degree (CD) of star-shaped L-lactide (L-LA) and D,L-lactide (D,L-LA) oligomers with different structures of arms. Raman spectroscopy was applied for the first time to analyze the structure of these oligomers. They differed in the number of arms (three or six) and the degree of polymerization (DP) of each arm (about 25 or 100). L-LA oligomers were studied in two forms: semicrystalline and amorphous. It was shown that the Raman method for evaluation of the CD, previously proposed for pure L-polylactide (L-PLA), linear L-LA oligomers, and L-LA copolymers with ε-caprolactone (CL), is applicable to the star-shaped L-LA oligomers. The CD values of the star-shaped L-LA oligomers evaluated by Raman spectroscopy and X-ray diffraction analysis were in a good agreement. Raman analysis showed that the CD of star-shaped L-LA oligomers increased with decreasing arm number and increasing arm length. These results demonstrate that Raman spectroscopy is an effective tool for monitoring the CD of star-shaped L-LA oligomers, including operative control of CD during annealing.

## 1. Introduction

### 1.1. Polylactide-Based Materials and Their Applications

Polylactide (PLA) is a biocompatible, biodegradable, non-toxic, thermoplastic polyester derived from lactic acid, or lactide (LA). PLA is an environmentally friendly polymer because of several facts. Firstly, the lactic acid used for its production is obtained by fermentation of carbohydrates, which in turn are obtained from relatively cheap and annually renewable plant biomass. Furthermore, obtaining raw materials for PLA from agricultural byproducts and waste is a promising approach. Secondly, PLA decomposes under natural conditions into carbon dioxide and water without harming the environment.

PLA is recyclable, and there are mechanical and chemical processing methods currently used for this polymer. In particular, chemical depolymerization converts PLA into high-purity lactic acid suitable for repolymerization. In cases where recycling is not possible due to contamination with organic or hazardous waste, PLA can be safely disposed of by industrial composting.

Currently, PLA is one of the most popular and promising of environmentally friendly polymers. PLA-based materials are already widely used in various fields, including as composites for 3D printing, for the production of various eco-friendly food packaging (containers for cold drinks and meals, bags, films, coffee capsules), disposable tableware, and non-woven products (medical clothing, personal hygiene products, tea bags) [1].

In addition, PLA is one of the promising candidates for agricultural applications among the environmentally friendly polymers available today [2,3]. PLA-based compositions are used for pre-sowing seed treatment, as well as for producing the covering and mulching materials [2,4]. The technology of planting grain seeds in a ribbon has also been developed [2]. Such an approach can help to protect seeds from the effects of aggressive environmental factors and create a necessary microclimate for favorable germination of seeds and plant development [2]. A coating material based on PLA and starch was developed for slow-release fertilizers using a spraying technique [5]. Nanoscale sustained-release urea fiber materials were successfully fabricated by coaxial electrospinning by encapsulating urea inside PLA fibers [6].

However, the most important application of PLA-based materials is the manufacture of medical products, including resorbable surgical sutures, implants, scaffolds, and fasteners for surgery and orthopedics. A separate area of application is the creation of nanoparticle carriers for targeted drug delivery with prolonged release of the active substance, as well as the creation of tissue-engineered constructions [7,8,9].

The properties and, consequently, potential applications of PLA-based materials are determined by their structure, which in turn depends on the synthesis and post-processing conditions. A key advantage of using PLA-based materials is the ability to specifically and broadly control their physicochemical properties by varying the synthesis conditions and by using various types of post-processing such as annealing and deformation. Synthesis conditions in this context can reflect modifying end groups; creating composites, copolymers, and blends; and changing molecular weight, enantiomeric chain composition, degree of branching, and molecular architecture. Depending on the chain microstructure and crystallization conditions, PLA can form various types of crystalline structures. Controlled annealing and cooling can change the crystallinity degree (CD) of L-PLA from 0 to 85%.

Both linear and branched LA oligomers are widely used. The architecture, size, and enantiomeric composition of LA oligomer chains determine their properties, degradation mechanism and rate, and, consequently, potential applications. The content of terminal functional groups in branched LA oligomers is higher than in linear high-molecular-weight PLA. This provides them with higher solubility and enables modification of the end groups, for example, to bind biologically active ligands, drugs or hydrophilic macromolecules to impart amphiphilic properties [10].

In medicine and pharmaceuticals, LA oligomers are used to create tissue-engineered constructions and targeted drug delivery systems with a predetermined drug release profile. In recent years, the development of additive manufacturing has opened up the prospect of creating biodegradable implants of complex shapes based on LA oligomers, including patient-specific implants [11]. Such implants are in high demand: as an example, methacrylated LA oligomers can be used as photocurable compositions for 3D printing of biocompatible and biodegradable medical devices.

Modified LA oligomers are used as additives to high-molecular-weight PLA in order to improve its mechanical properties [12] and regulate its degradation mechanism.

### 1.2. Studies on Star-Shaped Lactide Oligomers

Star-shaped LA oligomers are one type of branched LA oligomers. They have a structure in which several chains (arms) originate from a common center. Branched LA oligomers, including star-shaped ones, are obtained by ring-opening polymerization of LA in the presence of a co-initiator. The architecture of the synthesized oligomer depends on the structure of the chosen co-initiator [13,14,15]. The length of the arms is determined by the ratio of the molar contents of monomer and the co-initiator in the reaction mixture.

Currently, the most commonly used methods for analyzing the structure and properties of PLA-based materials include differential scanning calorimetry (DSC) [13,15,16], X-ray diffraction (XRD) analysis [13,17], gel permeation chromatography (GPC) [13,15], Fourier transform infrared (IR) spectroscopy [14,18,19,20], and nuclear magnetic resonance (NMR) spectroscopy [13,15]. It should be noted that each of these methods determines (directly or indirectly) only a part of the material’s structural characteristics. Therefore, several research methods are typically used simultaneously.

Due to many advantages (such as high information content; non-destructive nature; lack of need for sample preparation; high speed and ease of implementation of the techniques, including in a portable format), Raman spectroscopy is a very convenient method for solving many problems. It is widely used in the development of polymeric materials, quality control, and in studying the dependence of properties and the degradation mechanism on the original structure of the sample and the method of its preparation. Raman spectroscopy enables the analysis of the structure of complex-shaped objects with micron-level spatial resolution across the surface. This is in demand, for example, when analyzing implants or tissue-engineered constructions. Raman spectroscopy can be used for liquid and solid samples, aqueous dispersions, and hydrogels. Routine Raman analysis of samples can be fully automated and does not require highly qualified personnel. It is significantly less labor intensive than XRD analysis, GPC, and NMR spectroscopy. Raman spectroscopy also enables online monitoring of material structure, including during the manufacturing process.

Currently, Raman spectroscopy methods for quantitative structural analysis of materials based on LA oligomers are practically undeveloped. Moreover, this method has not been used at all for studying star-shaped LA oligomers. Interest in such studies has recently revived, primarily due to the development of new methods for synthesizing PLA-based materials and significant advances in the development of equipment for recording Raman spectra. However, this method is still rarely used in routine studies of PLA-based materials. Nevertheless, the Raman spectra of these materials can potentially be used to determine the chemical composition and CD of the material, the content of various end groups, and to estimate the molecular length in the case of short oligomers [21].

An analysis of published Raman spectroscopy data on PLA and PLA-based materials allows us to conclude that the Raman spectra of these materials depend on structural characteristics such as the CD [22,23,24], stereoregularity and chain packing order [23], the length of monomer unit sequences [17,25], molecular orientation [24,26,27,28], and comonomer content in the case of copolymers [29].

Among the most important publications on PLA Raman spectroscopy, it is worth noting the study [22], which proposed using the spectral range of the C=O stretching vibrations from 1700 to 1800 cm^−1^ to determine the CD of PLA. In this range, splitting is observed in the Raman spectrum of highly crystalline PLA, and this splitting is absent in the spectrum of amorphous PLA. However, this splitting is clearly expressed only for samples whose CD exceeds 50%. Thus, using the Raman lines of the C=O stretching vibrations to determine the CD of PLA is difficult for low-crystalline samples.

Furthermore, this spectral range cannot be used to analyze copolymers and blends in which the second component molecules (e.g., poly(ε-caprolactone) or polyglycolide) contain C=O bonds. That is because in this case, this range is influenced not only by the CD of the PLA regions but also by the composition of the copolymer or blend. These copolymers and blends are of particular interest for practical applications.

Recent studies [21,30] have proposed a method for determining the CD of L-PLA regions in L-PLA-based materials using Raman spectra. Several series of samples (pure L-PLA, linear L-LA oligomers, and L-LA copolymers with ε-caprolactone (CL)) were analyzed. It was shown that the ratio of peak intensities of the L-PLA Raman bands at 411 and 874 cm^−1^ is a linear function of the CD of the L-PLA regions, determined by XRD analysis. The Raman band at 411 cm^−1^ corresponds to the scissoring vibrations of the O–C(H)C(H_3_) bonds of PLA units, and the Raman band at 874 cm^−1^ corresponds to the symmetric stretching vibrations of the C−O−C bonds in the backbone of L-PLA unit sequences. This technique was tested for samples with a CD of PLA from 0 to 86%.

Studies [21,31] have also investigated the dependence of the Raman spectra of linear LA oligomers on their length. Density functional theory calculations of the structure and Raman spectra of linear L-LA oligomers in the 10_3_ helix conformation [31] demonstrated a weak dependence of the Raman spectra on molecular length. This conformation is characteristic of the most stable α-modification of PLA with an orthorhombic crystal cell. When the number of monomer units varied from 5 to 12, the calculated Raman spectra did not undergo significant changes. The same result was confirmed experimentally for linear L-LA and D,L-LA oligomers [21].

However, the study [21] demonstrated that, in the case when linear LA oligomers are synthesized using a co-initiator, the Raman bands of the co-initiator can be used to estimate the oligomer length. The results of the study [31] show that the intensity of the Raman lines in the calculated spectra of LA oligomers increases linearly with an increase in the number of the monomer units. A part of the co-initiator molecule forms the central fragment of the synthesized oligomer molecule and remains unchanged with increasing oligomer length. Thus, the Raman bands of the reaction co-initiator can serve as an internal reference when analyzing the bands of PLA monomer units.

In the study [21], it was shown that using the ratio of the Raman band intensities of PLA and the co-initiator (which in this case was 1,12-dodecanediol) makes it possible to estimate the degree of polymerization (DP) of linear LA oligomers up to 40 PLA monomer units. In particular, it was shown that the dependence of the intensity ratio of the Raman bands of 1,12-dodecanediol at 2850 cm^−1^ and PLA units at 2947 cm^−1^ on the DP is very suitable for assessing the DP of LA oligomers, since it is independent of the enantiomeric composition, physical state, and CD of the LA oligomers. The PLA Raman band at 2947 cm^−1^ corresponds to the symmetrical stretching vibrations of CH_3_ groups, and the 1,12-dodecanediol band at 2850 cm^−1^ to the symmetrical stretching vibrations of CH_2_ groups. The results of determining the DP of the oligomers by Raman spectroscopy are in excellent agreement with the data obtained using NMR spectroscopy.

Thus, star-shaped LA oligomers are promising materials for medical applications, and Raman spectroscopy has not been used to analyze such oligomers. Since Raman spectroscopy has proven itself effective in determining the CD for linear PLA-based materials, it makes much sense to test the applicability of this technique to determining the CD of star-shaped L-LA oligomers and to monitor changes in their Raman spectra depending on the number and length of arms. This will expand the scope of application of the previously proposed Raman method for molecules with a linear structure to molecules with branched molecular architecture.

Thus, in this work, for the first time, we applied Raman spectroscopy to the analysis of star-shaped L-LA and D,L-LA oligomers with varying numbers and lengths of arms. Raman spectroscopy was used as the primary method of the study, and XRD analysis was used as a supplementary method.

## 2. Materials and Methods

### 2.1. Samples

**Figure 1:**
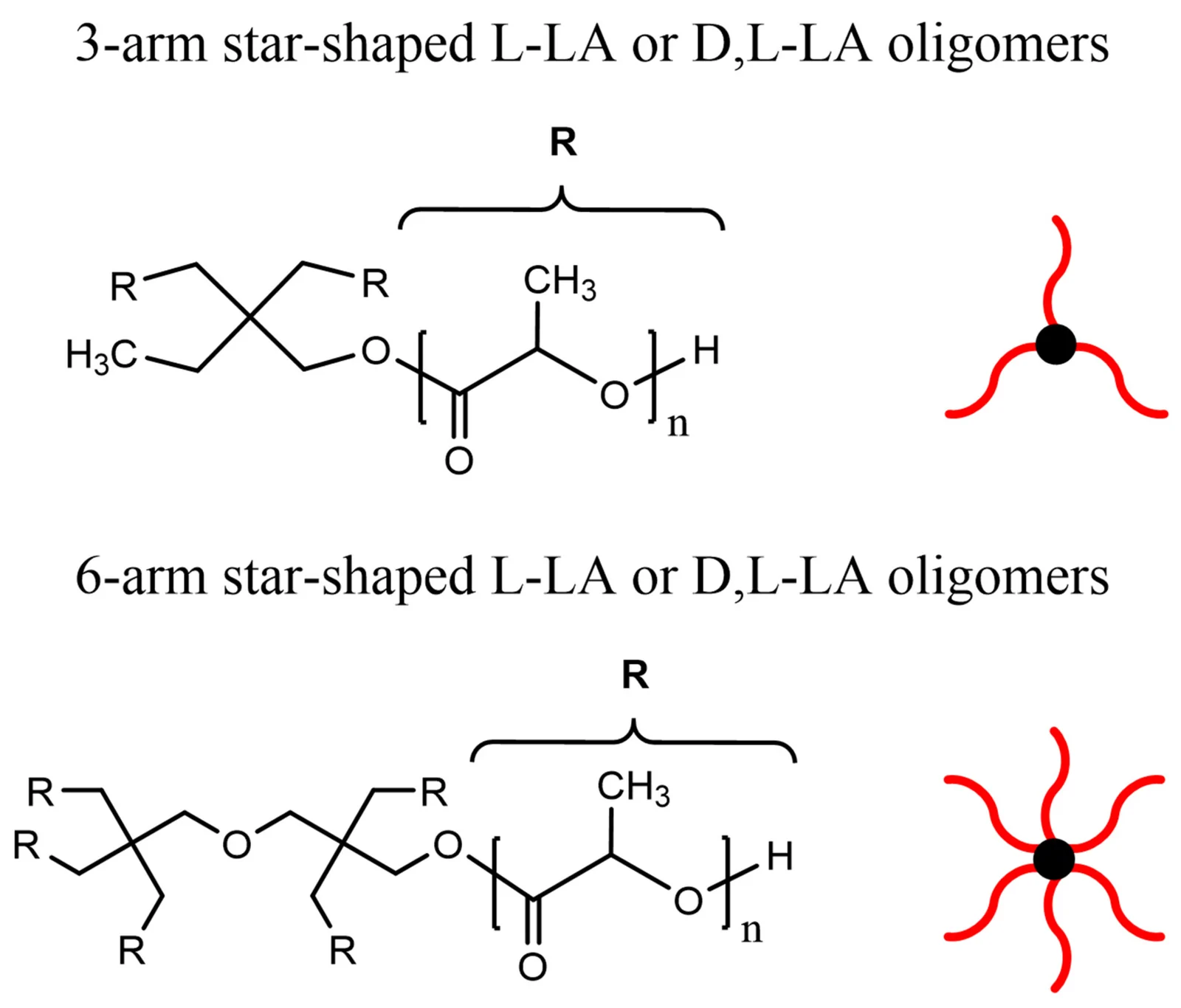
Structural formulas and schematic representations of structure of three-arm and six-arm star-shaped L-LA and D,L-LA oligomers.

In this study, we investigated samples of star-shaped L-LA and D,L-LA oligomers with 3 and 6 arms. The arm lengths were approximately 25 or 100 PLA monomer units (lactic acid units). Their structural formulas and schematic representations of structure are shown in Figure 1. The synthesis procedures and comprehensive characterization of these star-shaped oligomers of LA have been reported in detail in [13,32]. Their molecular architectures and molecular characteristics were established using complementary analytical techniques, including NMR spectroscopy, size exclusion chromatography (SEC)/GPC with triple detection, matrix-assisted laser desorption/ionization (MALDI) mass spectrometry, DSC, and XRD analysis. Therefore, in the present study, these previously characterized oligomers were used as well-defined model systems to investigate their Raman spectra as well as their dependence on the CD.

The oligomers were synthesized by one-step ring-opening polymerization of LA. Three-arm oligomers were synthesized in the presence of the reaction co-initiator trimethylolpropane, and six-arm oligomers were synthesized in the presence of the reaction co-initiator dipentaerythritol (Figure 1). Thus, the oligomers differed in the number and length of arms and the enantiomeric composition of the chains.

Two series of 200 μm thick films were prepared for the L-LA oligomers. In the first series, the films were amorphized by rapid cooling from the melt. The CD of these samples was close to 0%. The second series of the samples was annealed for 6 h in order to form a high proportion of the crystalline fraction. The annealing temperature was below the melting point of PLA and was selected in the range from 90 to 125 °C depending on the length of the oligomer arms [13].

The D,L-LA oligomers were amorphous due to the statistical distribution of L- and D-units in the chain, so one series of 200 μm thick amorphous films was prepared for these oligomers [32].

Table 1 shows the CD of the semicrystalline series of L-LA oligomers, measured specially for this work by XRD analysis [13]. It was shown in [13] that the α-modification [33,34] of the crystalline phase forms in L-LA oligomers. This modification is the most common for L-PLA and has an orthorhombic or pseudo-orthorhombic crystal lattice [35]. The unit cell of the α-form of L-PLA contains two molecules in the conformation of left-handed antiparallel helices 10_3_.

Four samples—amorphous P3L-25a, semicrystalline P6L-25c (CD = 28%) and P6L-100c (CD = 69%), and amorphous P6DL-100—were further annealed in a Utenos Elektrotechnika SNOL 67/350 furnace (Lithuania) at 75 °C for 30, 60, 90, and 120 min. After annealing, the samples were cooled in air at room temperature (~25 °C).

For comparison, commercial granular samples of PLA stereoisomers (Corbion, Amsterdam, The Netherlands) were analyzed: L-PLA and D,L-PLA with a 50:50 ratio of D- and L-lactic acid units and a statistical distribution of the units along the chain. The CD of the samples, determined by XRD analysis, was 86% for L-PLA and 0% for D,L-PLA. Additionally, an amorphous L-PLA film was produced from the same L-PLA granules by rapid cooling from the melt.

Furthermore, for comparison, the Raman spectra of the starting monomers—commercial powder samples of L-LA and D,L-LA (99% purity, Corbion, The Netherlands)—were analyzed.

### 2.2. Raman Spectroscopy

Raman spectra were recorded at room temperature (~25 °C) using a Senterra II Raman confocal microscope (Bruker Optics, Billerica, MA, USA). The spectra were recorded at 180° scattering and with a spectral resolution of 1.5 or 4 cm^−1^. In this study, the excitation radiation had wavelengths of 532 or 785 nm. For a wavelength of 532 nm, a Nd:YAG laser (Bruker Optics, USA) with an output power of 25 mW was used, and for a wavelength of 785 nm, a diode laser (Bruker Optics, USA) with an output power of 100 mW was used. Both excitation and scattered radiations were focused by an objective with a magnification of 20× (N.A. 0.40). The laser spot diameter on the sample surface was 10 μm at an excitation wavelength of 532 nm and 12 μm at an excitation wavelength of 785 nm.

The spectrum acquisition time was determined individually for each sample to ensure a good signal-to-noise ratio; however, for all the samples, the spectrum acquisition time did not exceed a few minutes.

In order to ensure that the sample structure was homogeneous, Raman spectra were recorded and compared at five different points on the surface of each sample. Analysis revealed that for all the samples, the spectra measured at different points on the surface were almost identical. Therefore, the spectrum obtained by averaging the spectra recorded at different points on the surface was used for analysis.

In order to verify that the sample structure did not change during the spectral acquisition process due to heating by the laser beam, spectra were recorded several times for each sample at the same point on the surface at intervals of several minutes. All these spectra were identical, indicating that, under the chosen spectrum recording conditions, the sample structure was not altered by heating by the laser radiation. The CD of the same L-LA oligomer sample, calculated from spectra measured at five points on the surface, and calculated from a series of measurements at one point but at different time intervals, coincided within 5%.

For all Raman spectra recorded with an excitation wavelength of 785 nm, the fluorescence background was minimal and did not interfere with the analysis of the Raman bands. However, in the case of the 532 nm excitation wavelength, a noticeable fluorescence background was observed. This background was likely due to impurities remaining after synthesis.

Given that a high-fluorescence background can affect the results of Raman line analysis, the main conclusions of this study are based on the analysis of Raman spectra recorded with excitation wavelength of 785 nm. The results obtained with an excitation wavelength of 532 nm were used only for comparison with the results obtained with an excitation wavelength of 785 nm.

Since sufficiently intense and well-resolved lines were selected for analysis, the measurement error of the peak intensity ratio of the Raman lines in the spectra recorded with an excitation wavelength of 785 nm was estimated at 5–7%.

Furthermore, Raman spectra recorded with a spectral resolution of 1.5 cm^−1^ were used for the main analysis, as at this resolution, splitting of some lines is observed in the Raman spectra of highly crystalline L-PLA, particularly lines near 400 and 1750 cm^−1^. Spectra recorded at a spectral resolution of 4 cm^−1^ were used for comparison only, as no line splitting was observed in this case.

In this paper, all experimental Raman spectra are presented after background subtraction and normalization to the peak intensities of the Raman lines. No additional mathematical processing of the spectra was performed.

### 2.3. XRD Analysis

X-ray diffraction analysis was performed at the BioMUR 3.5 station (Moscow, Russia) of the Kurchatov synchrotron in the transmission geometry using the solid-state holder [36,37]. The radiation source was a 1.7 T bending magnet with an energy of 8.8 keV (1.445 Å), a resolution of dE/E of 10^−3^, and a photon flux of 10^9^. The beam size on the sample was 0.5 × 0.3 mm, and the positioning of the beam on the sample was performed using the video camera aligned along the beam direction and a motorized slide with accuracy of movements within 10 μm. A Dectris Pilatus 1M two-dimensional detector was used to record diffraction patterns. The sample-to-detector distance was 150 mm, and background scattering was subtracted before subsequent processing. Silver behenate and NaC(Na_2_Ca_3_Al_2_F_14_) were used as calibration standards. The range of backscattering vector q values was 0.8–33 nm^−1^, and the exposure time was 300 s. The obtained scattering patterns were processed using the Fit2D and OriginPro software (v2018) packages. The CD was calculated from the obtained wide-angle X-ray scattering (WAXS) curves using the standard procedure of fitting both sharp crystalline reflections with Pearson shapes and amorphous halo with wide Gaussian shape after background subtraction. The CD value of oligomers of L-LA equals the ratio of area $S_{cryst}$ of crystalline phase reflections to the sum of $S_{cryst}$ and $S_{amorph}$, which is the area of the amorphous halo:

(1) $$\chi =\frac{S_{cryst}}{S_{cryst}+S_{amorph}}\cdot 100\%$$

The error in CD determination by the XRD method was estimated at 2–5%.

## 3. Results and Discussion

**Figure 2:**
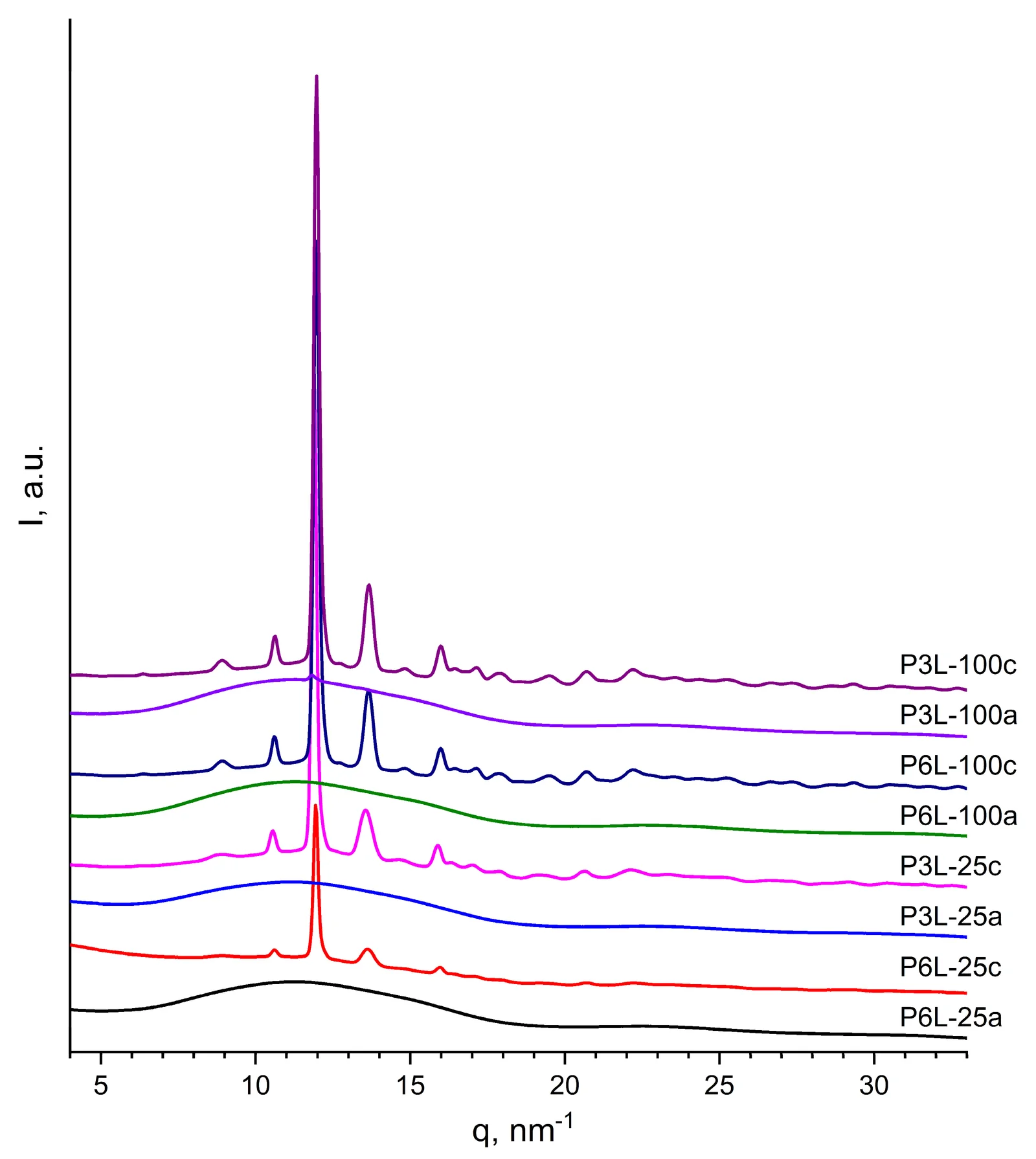
WAXS curves of star-shaped L-LA oligomers.

WAXS curves were obtained at room temperature (Figure 2). Two main sharp crystalline peaks were observed for star-shaped oligomers of L-LA with DP of 25 and 100 at q values of around 11.9 and 13.6 nm^−1^. These two peaks are attributed to the diffraction from (110)/(200) and (203)/(113) lattice planes of the α-form of poly(L-lactide). Other reflections clearly observed on the WAXS profiles also confirm the formation of the α-crystalline phase [34]. It was found that the amorphized L-LA oligomers P3L-25a, P3L-100a, P6L-25a, and P6L-100a are in a close to completely amorphous state (Table 1), since no crystalline diffraction peaks were observed (or they were of very low intensity).

**Figure 3:**
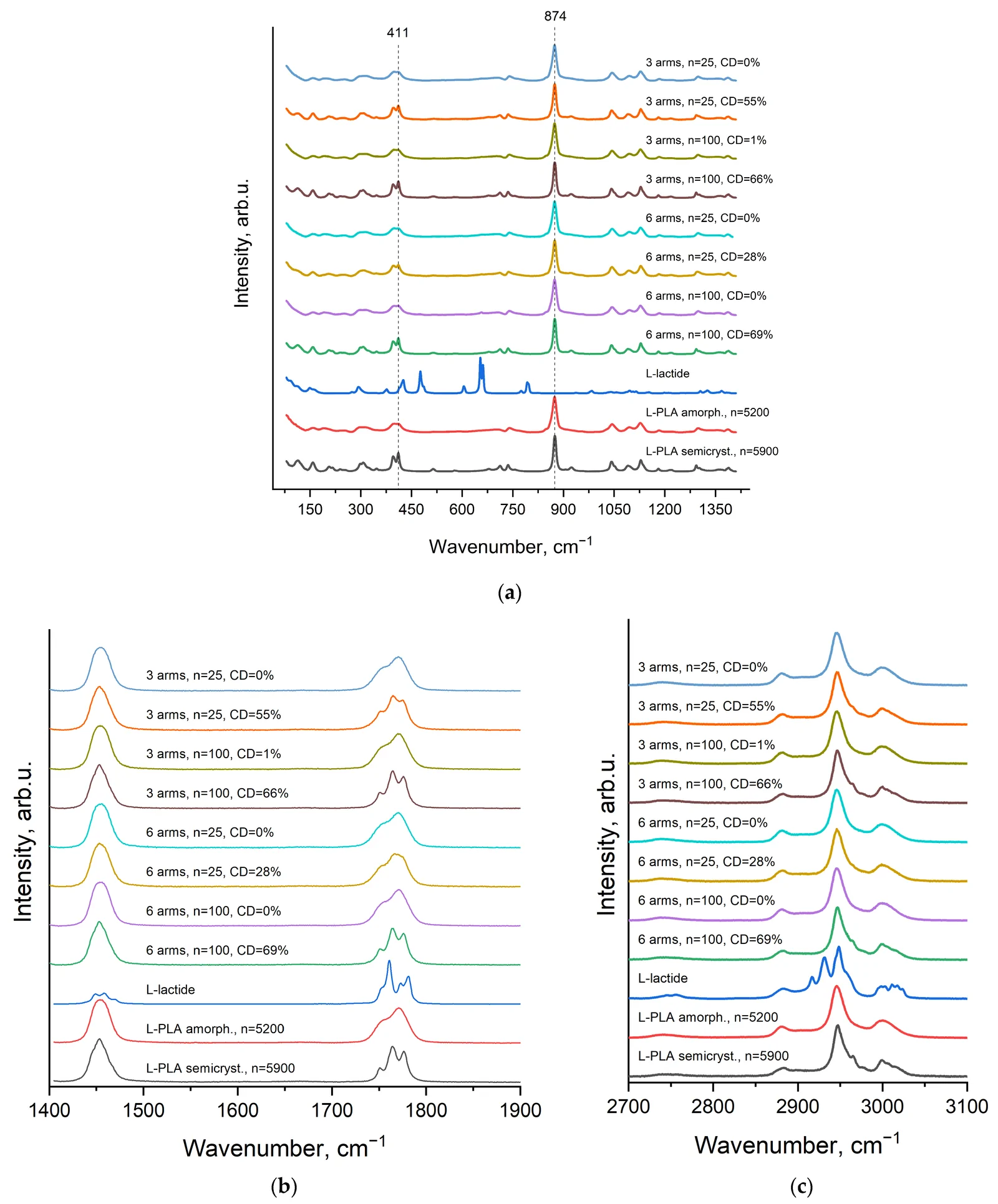
Raman spectra of star-shaped L-LA oligomers in three spectral ranges ((**a**) 80–1410 cm^−1^; (**b**) 1400–1900 cm^−1^; (**c**) 2700–3100 cm^−1^), recorded using excitation radiation with a wavelength of 785 nm and a spectral resolution of 1.5 cm^−1^. For comparison, the Raman spectra of L-LA and L-PLA with CD equal to 0 and 86% are shown.

**Figure 4:**
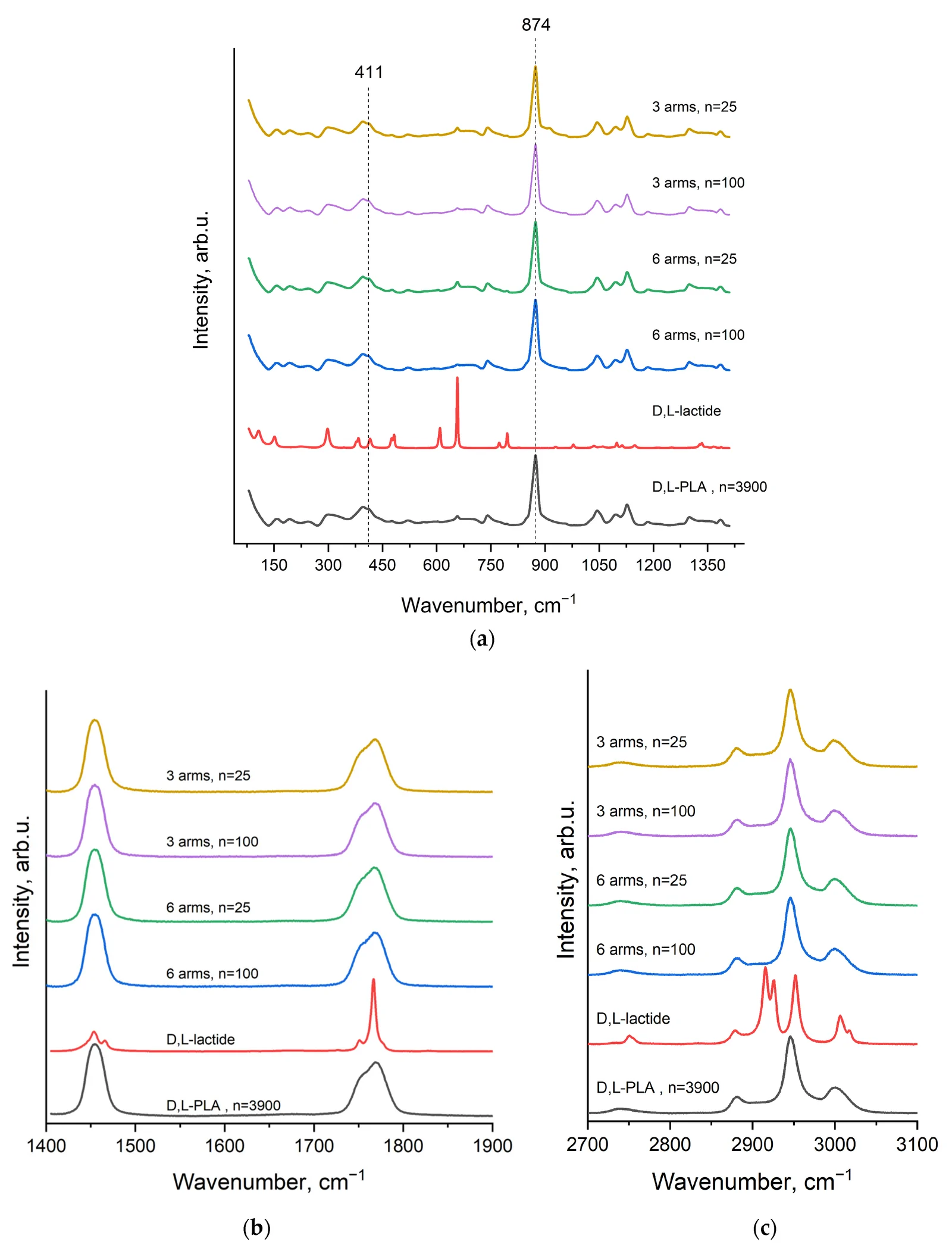
Raman spectra of star-shaped D,L-LA oligomers in three spectral ranges ((**a**) 80–1410 cm^−1^; (**b**) 1400–1900 cm^−1^; (**c**) 2700–3100 cm^−1^), recorded using excitation radiation with a wavelength of 785 nm and a spectral resolution of 1.5 cm^−1^. The Raman spectra of D,L-LA and amorphous D,L-PLA are shown for comparison.

**Figure 5:**
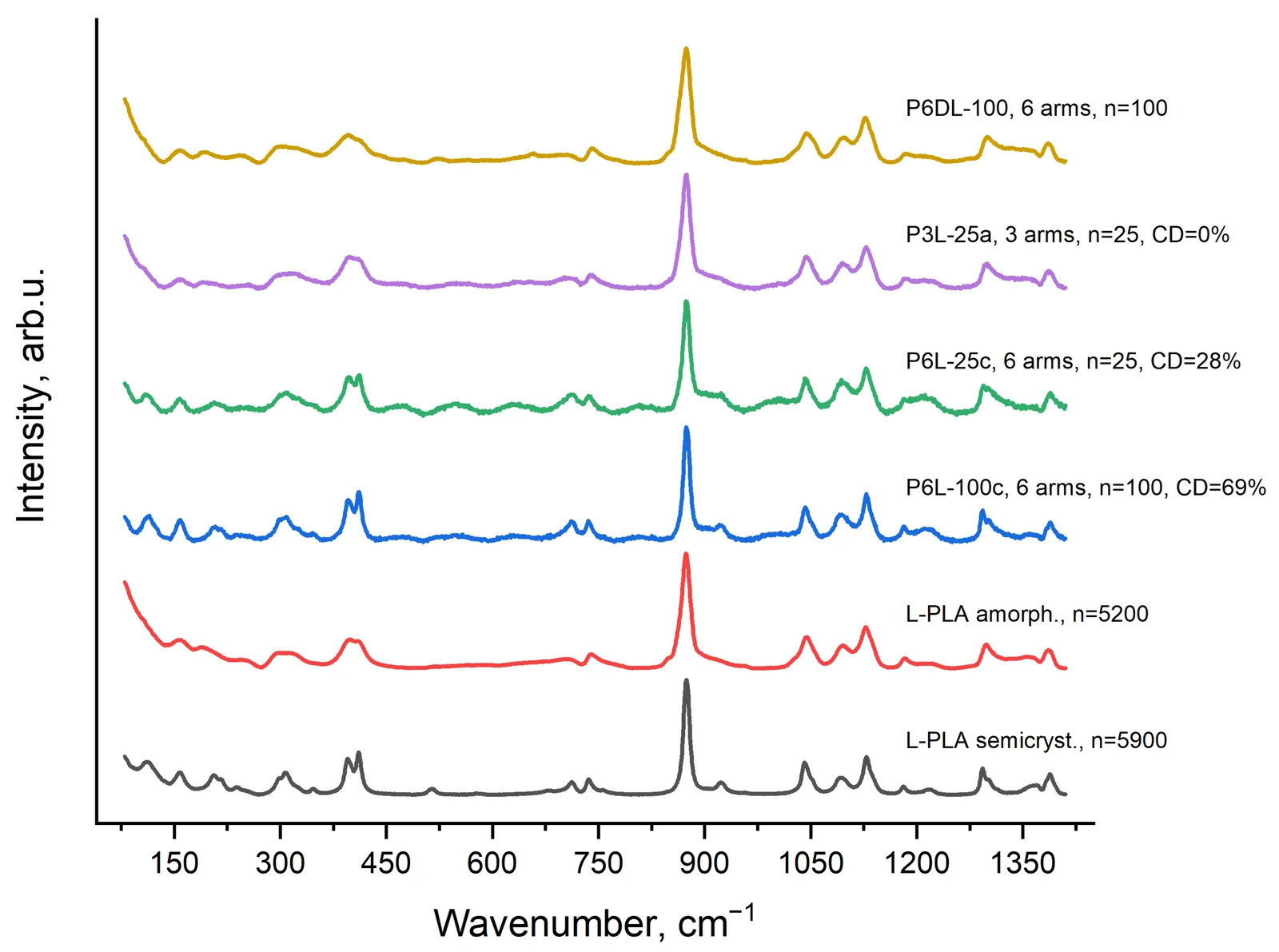
Raman spectra of star-shaped L-LA and D,L-LA oligomers in the range of 80–1410 cm^−1^ recorded using excitation radiation with a wavelength of 532 nm and a spectral resolution of 1.5 cm^−1^. The Raman spectra of L-PLA with a CD of 0 and 86% are shown for comparison.

Figure 3 shows the Raman spectra of the star-shaped L-LA oligomers in three spectral ranges, recorded with laser excitation with a wavelength of 785 nm. The Raman spectra of L-LA and L-PLA with a CD of 0 and 86% are shown for comparison. From here on, the figures show the CD of all the materials determined by XRD analysis, the DP of arms of the star-shaped oligomers measured by NMR spectroscopy, and the DP of the amorphous and semicrystalline L-PLA (amorphous D,L-PLA in Figure 4 and Figure 5) measured by GPC [38]. From here on, intensities in the Raman spectra are normalized to the peak intensity of the most intense line in each spectral range under consideration.

The most noticeable difference with the change in CD of the L-LA oligomers is observed for the bands around 400 and 1750 cm^−1^. In particular, with increasing CD, the splitting of these bands becomes more pronounced, the intensity of the doublet at about 400 cm^−1^ increases, and the intensity of the high-wavenumber component of the doublet increases faster than the intensity of the low-wavenumber component. These patterns are completely consistent with the patterns observed with increasing CD of L-PLA (L-PLA regions) in the Raman spectra of L-PLA, linear L-LA oligomers, and random copolymers of L-LA and CL [21,30].

For the amorphous L-LA oligomers with different numbers and lengths of arms, the Raman spectra are almost identical. This result is consistent with the results of the Raman study of amorphous linear L-LA oligomers [21].

Figure 4 shows the Raman spectra of the star-shaped D,L-LA oligomers in three spectral ranges, recorded using excitation radiation with a wavelength of 785 nm. The Raman spectra of D,L-LA and amorphous D,L-PLA are shown for comparison.

All the D,L-LA oligomers are amorphous due to the statistical distribution of L- and D-units. No significant differences are observed in the spectra of D,L-LA oligomers, as in the spectra of amorphous L-LA oligomers, and this experimental result is consistent with the results of the Raman study of linear D,L-LA oligomers [21].

Thus, it can be concluded that the Raman spectra of the star-shaped L-LA and D,L-LA oligomers do not directly depend on the number, DP, and enantiomeric composition of the arms, but depend on the CD. This result is in excellent agreement with the results of the study of the linear L-LA and D,L-LA oligomers [21], for which it was also shown that the Raman bands of the oligomers weakly depend on the length of the oligomer and the enantiomeric composition of the chains, but significantly depend on the CD.

Figure 5 shows the most characteristic Raman spectra of the star-shaped L-LA and D,L-LA oligomers in the 80–1410 cm^−1^ range, recorded with laser radiation with a wavelength of 532 nm.

In general, there are no fundamental differences between the Raman spectra of the oligomers recorded using laser radiation with wavelengths of 532 and 785 nm. However, with a wavelength of 532 nm, a higher-fluorescence background is observed and the signal-to-noise ratio is significantly lower. The patterns observed in the Raman spectra of the oligomers recorded with excitation with a wavelength of 532 nm are completely consistent with those observed in the case of 785 nm wavelength. Specifically, the Raman spectra of the oligomers do not directly depend on the number, DP, and enantiomeric composition of the arms but are significantly dependent on the CD. The dependence on CD is most pronounced for the bands at 400 and 1750 cm^−1^.

As mentioned in Section 1, a method for determining the CD of L-PLA regions in L-PLA-based materials using Raman spectra was proposed in recent studies [21,30]. Several series of samples (pure L-PLA, linear L-LA oligomers, and L-LA copolymers with CL) were analyzed, and it was shown that the ratio of the peak intensities of the Raman bands of L-PLA units at 411 and 874 cm^−1^ is a linear function of the CD of L-PLA regions, obtained using XRD analysis.

**Figure 6:**
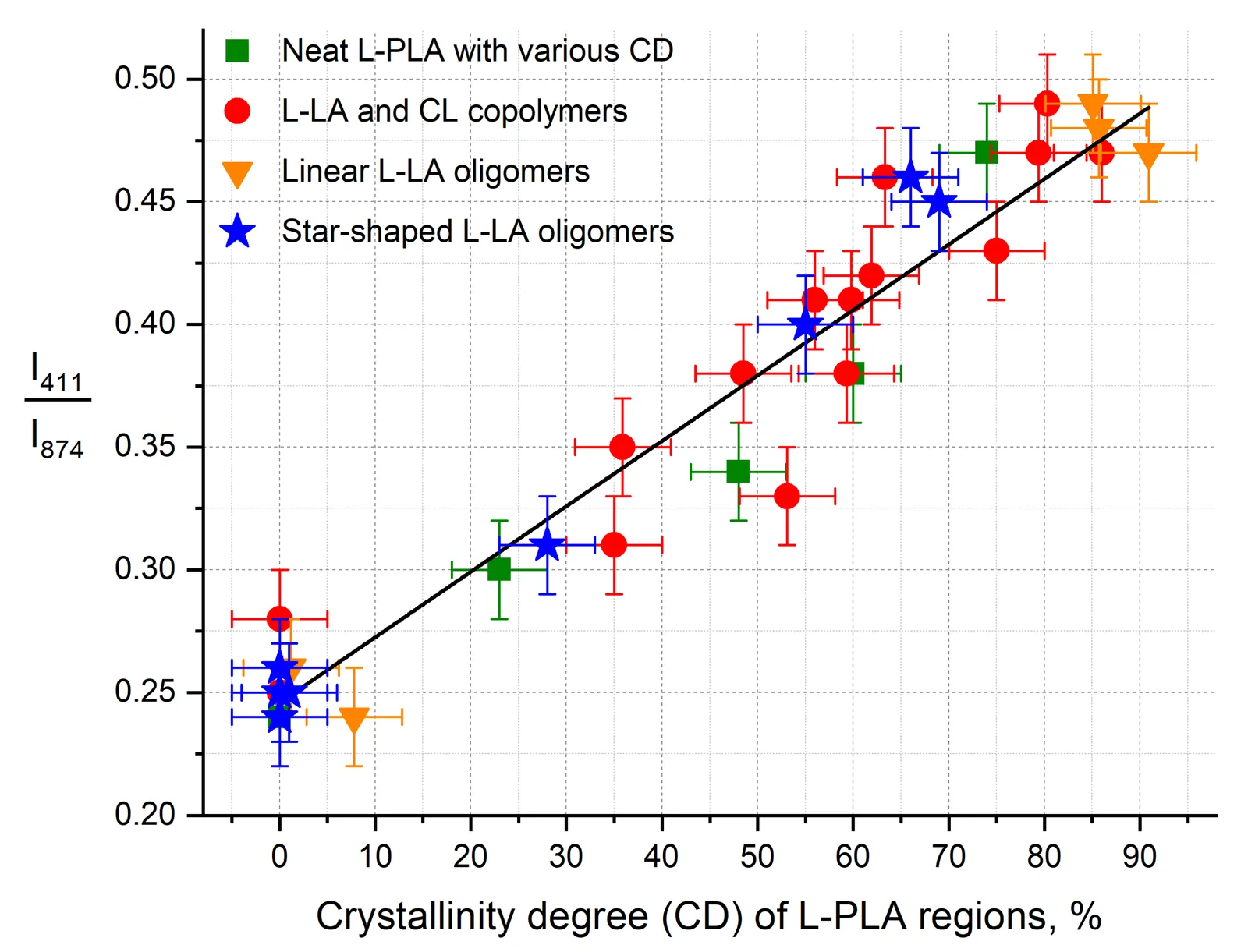
Dependence of the ratio of the peak intensities of Raman bands of L-PLA units at 411 and 874 cm^−1^ for the star-shaped L-LA oligomers studied in this work in comparison with other samples from [21,30] on the CD obtained by XRD analysis.

One of the goals of this work was to test the possibility of determining the CD of star-shaped L-LA oligomers using Raman spectroscopy according to the method proposed in works [21,30]. Figure 6 shows the dependence of the ratio of peak intensities of the Raman bands of L-PLA units at 411 and 874 cm^−1^ for the samples from [21,30] and the star-shaped L-LA oligomers studied in this work. The linear dependence obtained in [21,30] has the form:

(2) $$\frac{I_{411}}{I_{874}}=0.0027\cdot CD+0.25.$$

As can be seen from Figure 6, the Raman spectroscopy method for assessing the CD of pure L-PLA, linear L-LA oligomers, and L-LA copolymers with CL is also well applicable to star-shaped L-LA oligomers.

**Figure 7:**
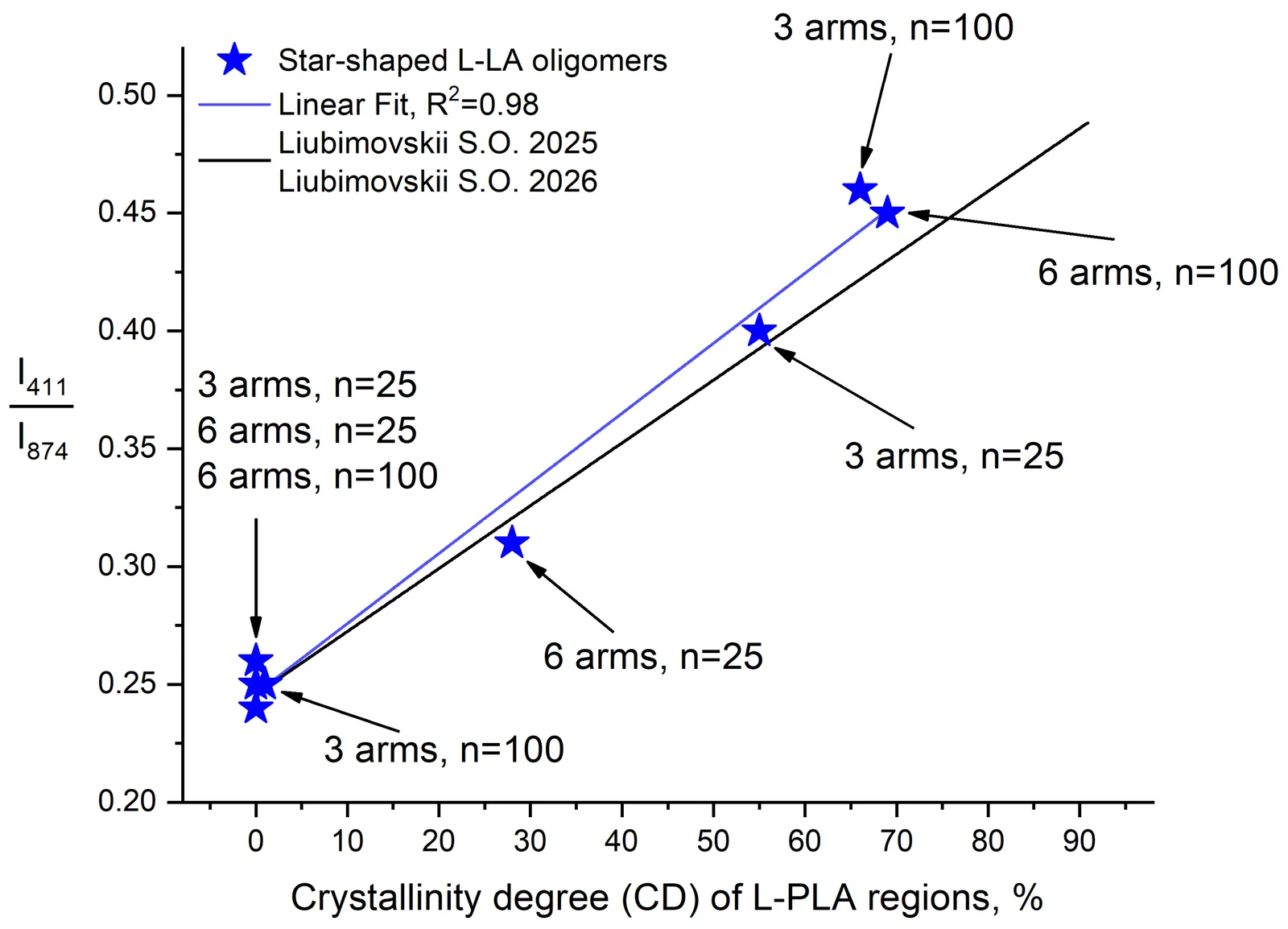
Dependence of the ratio of peak intensities of the L-PLA Raman bands at 411 and 874 cm^−1^ for samples from works [21,30] without indicating points for these samples and the star-shaped L-LA oligomers studied in this work on the CD obtained by the XRD analysis.

In order to more clearly illustrate the dependence of the CD of the star-shaped L-LA oligomers on the number and length of arms, Figure 7 shows the dependence of the peak intensity ratio of the Raman bands of L-PLA units with wavenumbers of 411 and 874 cm^−1^ for the samples that were studied in [21,30], without the points corresponding to the original experimental data for these samples. This dependence is very close to the dependence obtained separately for the star-shaped L-LA oligomers.

Notwithstanding the limited number of samples, it can be seen from Figure 7 that the CD of the star-shaped L-LA oligomers increases with a decrease in the number of arms and with an increase in the DP of the arms (this conclusion was made without taking into account specially amorphized films of the star-shaped L-LA oligomers). This result is expected, since the greater the number of arms is and the lower their DP value is, the more difficult it is for the oligomer molecules to form a crystal lattice. Note that the dependence of CD versus the arm number is less pronounced for the oligomers with n = 100. For long-arm oligomers, it is reasonable to assume that branching has less of an effect on this dependence.

In [13], it was noted that with an increase in the DP of the arms, an increase in the CD of L-LA oligomers was observed, which is associated with the formation of more perfect crystals, caused by a decrease in the influence of the end groups of the chains. This fact is known in the literature. At the same time, it was shown in [13] that the branching limits the segmental mobility of the oligomers (according to glass transition temperatures) and prevents the crystallization of poly(L-lactide) chains. Thus, both the Raman spectroscopy and XRD analysis data are in good agreement, and the methodology previously proposed for pure L-PLA, linear L-LA oligomers, and L-LA copolymers with CL is applicable to star-shaped L-LA oligomers.

**Figure 8:**
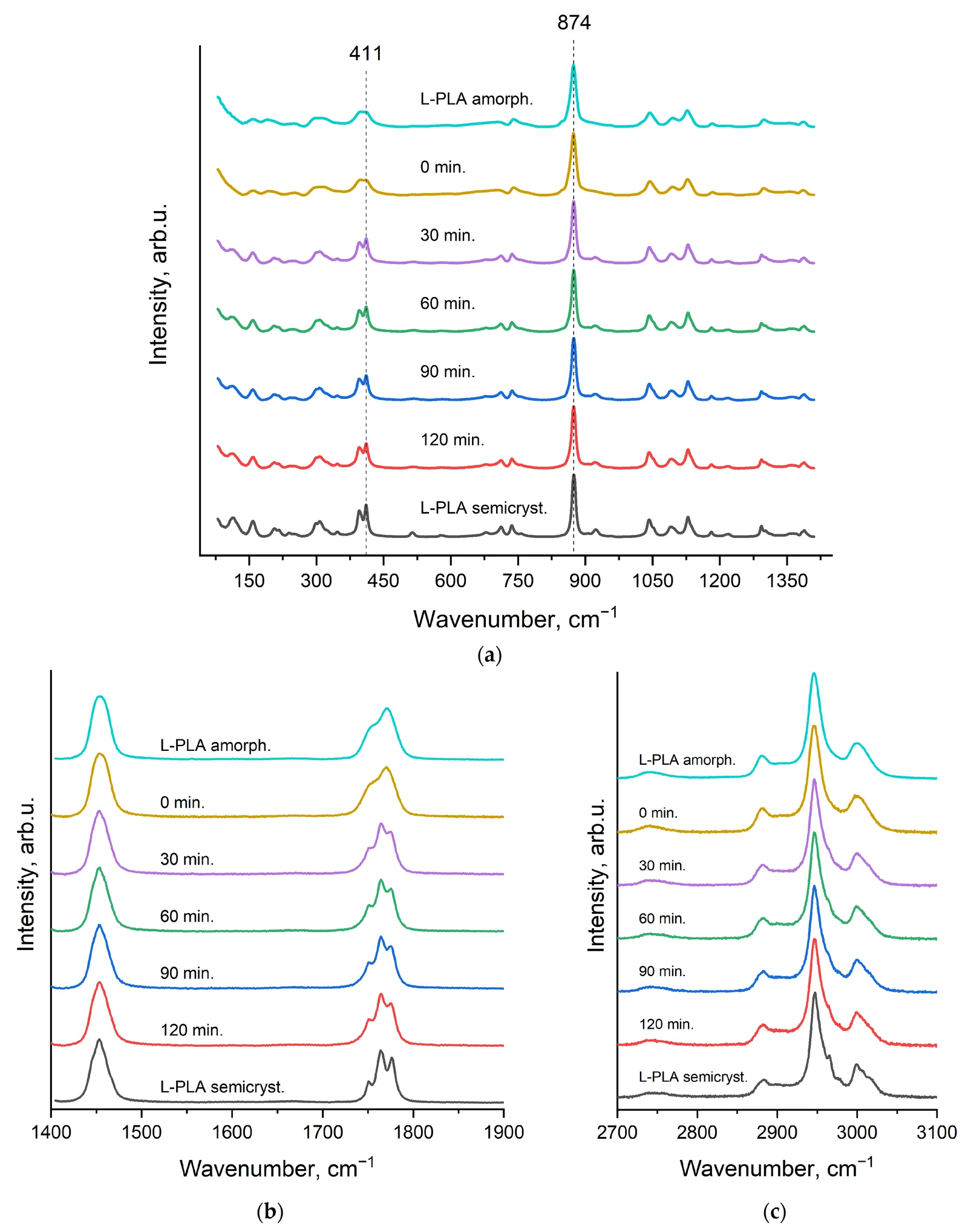
Raman spectra of the P3L-25a oligomer annealed for different time in three spectral ranges ((**a**) 80–1410 cm^−1^; (**b**) 1400–1900 cm^−1^; (**c**) 2700–3100 cm^−1^), recorded with excitation radiation with a wavelength of 785 nm and a spectral resolution of 1.5 cm^−1^. The Raman spectra of L-PLA with a CD of 0 and 86% are shown for comparison.

We now turn to a consideration of the results of studying the Raman spectra of the additionally annealed star-shaped LA oligomers. Figure 8 shows the Raman spectra of the P3L-25a oligomer annealed for different times in three spectral ranges, recorded with an excitation wavelength of 785 nm. For comparison, the Raman spectra of the original P3L-25a sample and commercial L-PLA samples with a CD of 0 and 86% are shown.

**Figure 9:**
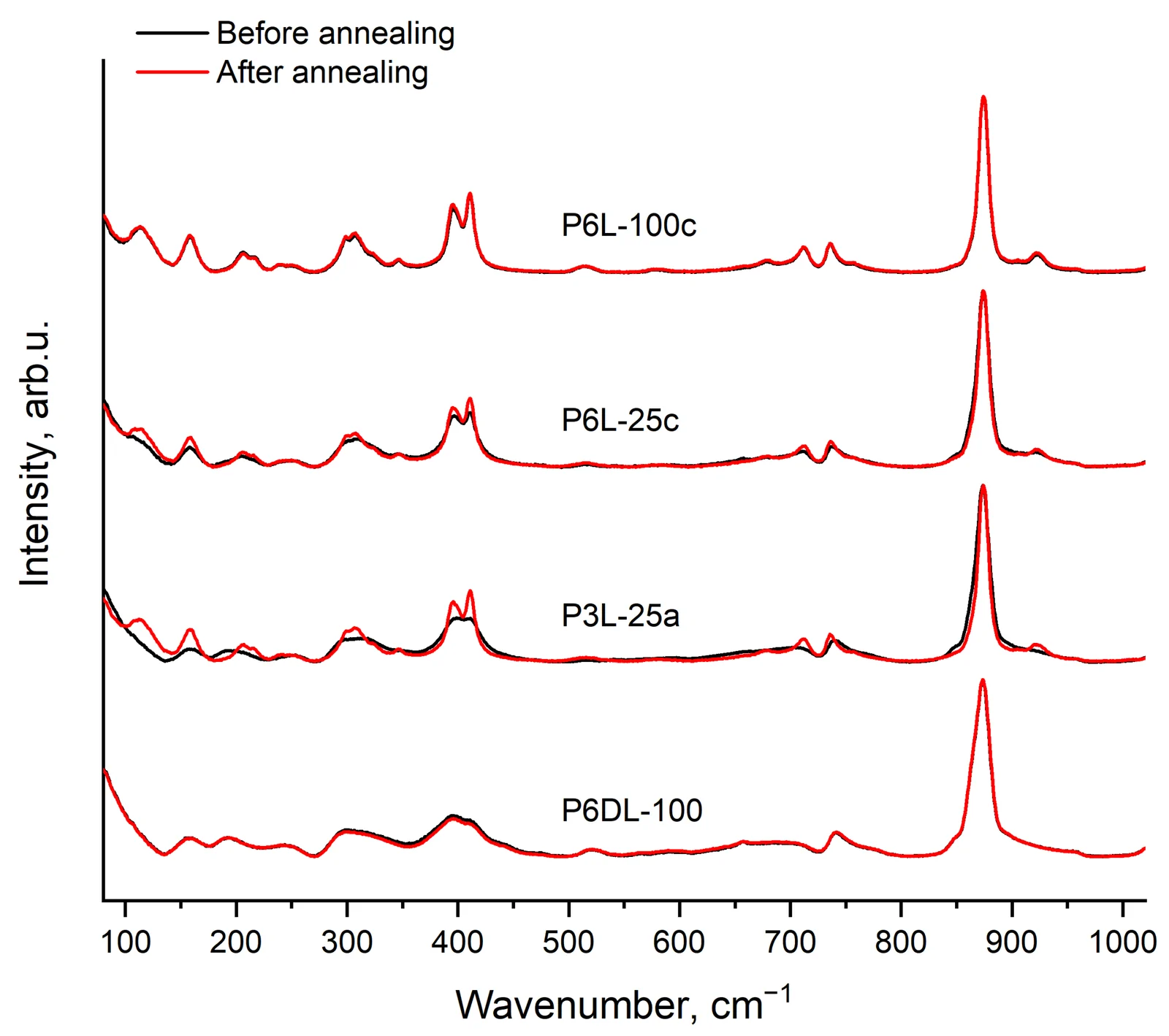
Raman spectra of star-shaped LA oligomers before annealing (black lines) and after annealing (red lines) for 120 min at 75 °C, recorded with excitation radiation with a wavelength of 785 nm and a spectral resolution of 1.5 cm^−1^.

Figure 9 shows the Raman spectra of four star-shaped LA oligomers before annealing and after annealing for 120 min in the range of 100–1000 cm^−1^, which is the most sensitive to changes in CD.

As can be seen from Figure 8 and Figure 9, the most pronounced changes in the oligomer’s Raman spectrum upon annealing are observed for the P3L-25a sample, which was initially amorphous but was capable of crystallization, unlike the P6DL-100 oligomer.

The Raman spectra were used to calculate the CD of the L-LA oligomers before and after annealing using dependence (2) (Table 2). As can be seen from Table 2, the CD values for the L-LA oligomers before annealing, obtained by Raman spectroscopy and XRD analysis, are in good agreement. As expected, annealing does not affect the structure of the D,L-LA oligomer, which is amorphous due to the statistical distribution of L- and D-units.

Analysis of the intensity ratio I_411_/I_874_ for the Raman spectra shown in Figure 8 shows that this ratio reaches 0.40 during 30 min of annealing and then remains unchanged within the measurement error of the Raman band intensity ratio. Thus, under these annealing and cooling conditions, the CD of the amorphous oligomer P3L-25a increases to 57% during 30 min of annealing and remains unchanged during further annealing for another 90 min.

As noted in Section 1, the C=O stretching vibration range in the PLA spectrum at about 1750 cm^−1^ is also sensitive to changes in the CD of this polymer. This fact is confirmed by the spectra shown in Figure 8b. As the CD of the P3L-25a oligomer increases during 30 min of annealing, the splitting of the lines around 1750 cm^−1^ becomes more pronounced and remains unchanged with further increase in annealing time. However, this range is not convenient to use for determining the CD of PLA due to the strong overlap of the bands and also because the lines of polymers that are often used in PLA-based medical materials, such as poly(ε-caprolactone)and polyglycolide, are observed in this range.

The spectral range shown in Figure 8c, corresponding to the region of the stretching vibrations of CH_3_ groups, depends weakly on the CD of PLA [21,30]. This allows this range to be used for evaluation of the chemical composition of PLA-based materials. For example, this range was used to determine the content of CL units in copolymers of LA and CL, and blends of PLA and poly(ε-caprolactone) [30].

## 4. Conclusions

In this work, star-shaped L-LA and D,L-LA oligomers were studied for the first time using Raman spectroscopy. The oligomers differed in the number of arms (three or six) and the DP of the arms (about 25 or 100). L-LA oligomers were prepared in two forms: semicrystalline and amorphous.

It was shown that the method for determining the CD by Raman spectra, previously proposed for pure L-PLA, linear L-LA oligomers, and L-LA copolymers with CL, is applicable to the star-shaped L-LA oligomers. It was found that the CD values of the star-shaped L-LA oligomers determined by Raman spectroscopy and XRD analysis are in a good agreement.

Raman analysis of the sample series showed that the CD of star-shaped L-LA oligomers increases with decreasing arm number and increasing arm length.

Raman spectroscopy demonstrated that annealing the amorphous or low-crystalline star-shaped L-LA oligomers at 75 °C for 30 min and then cooling the sample to room temperature increases the oligomer’s CD value. Annealing was found to have no effect on the structure of the star-shaped D,L-LA oligomer, which is amorphous due to the statistical distribution of L- and D-units.

Thus, Raman spectroscopy was demonstrated to be a convenient tool for monitoring the CD value of star-shaped L-LA oligomers, including such analysis during annealing.

## Figures and Tables

**Table 1:** Sample designations, DP of the arms, and CD of LA oligomers.

Sample Designations	Average DP of the Arms (NMR Spectroscopy [13,32])	CD, % (XRD Analysis)
L-LA oligomers		
P3L-25a	25	0
P3L-25c		55
P3L-100a	96 *	1
P3L-100c		66
P6L-25a	25	0
P6L-25c		28
P6L-100a	100	0
P6L-100c		69
D,L-LA oligomers		
P3DL-25	25	0
P3DL-100	100	0
P6DL-25	25	0
P6DL-100	100	0

* For simplicity, this DP value was rounded to 100 in the following text.

**Table 2:** DP of arms (data from NMR spectroscopy) and CD (data from XRD analysis and Raman spectroscopy) for star-shaped L-LA and D,L-LA oligomers before and after annealing.

Sample Designations	DP of Arms (NMR Spectroscopy [13,32])	CD Before Annealing, % (XRD Analysis [13,32])	I411/I874 Before Annealing	CD * Before Annealing, % (Raman Spectroscopy)	I411/I874 After Annealing	CD * After Annealing, % (Raman Spectroscopy)
L-LA oligomers						
P3L-25a	25	0	0.25	0	0.40	57
P6L-25c		28	0.31	22	0.39	52
P6L-100c	100	69	0.45	74	0.45	74
D,L-LA oligomer						
P6DL-100	100	—	0.21	—	0.18	—

* The CD of L-LA oligomers was calculated using Formula (2): $I_{411}/I_{874}=0.0027\cdot CD+0.25$.

## Data Availability

Data are contained within the article.

## Abbreviations

The following abbreviations are used in this manuscript:

CD	crystallinity degree
CL	ε-caprolactone
D,L-LA	D,L-lactide
DP	degree of polymerization
DSC	differential scanning calorimetry
GPC	gel permeation chromatography
IR	infrared
LA	lactide
L-LA	L-lactide
L-PLA	L-polylactide
N.A.	numerical aperture
NMR	nuclear magnetic resonance
PLA	polylactide
XRD	X-ray diffraction
